# Changes of alveolar bone dehiscence and fenestration after augmented corticotomy-assisted orthodontic treatment: a CBCT evaluation

**DOI:** 10.1186/s40510-019-0259-z

**Published:** 2019-02-18

**Authors:** Liangyan Sun, Lingjun Yuan, Bo Wang, Lina Zhang, Guofang Shen, Bing Fang

**Affiliations:** 1 Department of Orthodontics, Shanghai Stomatological Hospital, 1258 Fuxing Rd, 2nd Floor, Shanghai, 200000 China; 2 Department of Orthodontics, Ninth People’s Hospital, College of Stomatology, Shanghai Jiao Tong University School of Medicine, 500 Quxi Rd, 7th Floor, Shanghai, 200000 China; 3 Department of Oral & Cranio -Maxillofacial Science, Ninth People’s Hospital, College of Stomatology, Shanghai Jiao Tong University School of Medicine, 500 Quxi Rd, 4th Floor, Shanghai, 200000 China; 4 Department of Biostatistics, College of Stomatology, Shanghai Jiao Tong University School of Medicine, 227 South Chongqing Rd, Shanghai, 200000 China

**Keywords:** Augmented corticotomy-assisted orthodontic treatment, Dehiscence, Fenestration, CBCT

## Abstract

**Background:**

To evaluate the changes of alveolar dehiscence and fenestration after augmented corticotomy-assisted orthodontic treatment on cone-beam computed tomography (CBCT) compared with traditional pre-surgical orthodontics, both quantitatively and qualitatively.

**Methods:**

Two hundred and four anterior teeth from 17 skeletal class III malocclusions were divided into four groups. Groups G1 (upper teeth) and G3 (lower teeth), comprising 120 teeth, accepted traditional pre-surgical orthodontics; groups G2 (upper teeth) and G4(lower teeth), comprising 84 teeth, accepted augmented corticotomy-assisted pre-surgical orthodontics. The changes of alveolar bone dehiscence and fenestration of each tooth in all groups were evaluated with the help of CBCT.

**Results:**

Quantitative analysis for comparing both groups: For the upper teeth, *d*_1_ − *d*_0_ was different between both groups while *f*_1_ − *f*_0_ was not statistically different. For the lower teeth, *d*_1_ − *d*_0_ was statistically different between both groups while *f*_1_ − *f*_0_ was not statistically different. Qualitative analysis: For the teeth that had no dehiscence before treatment, G2 and G4 had a better transition than did G1 and G3. For those having dehiscence before treatment, G4 had a better transition than did G3. For teeth having no fenestration before treatment, there was no statistically significant difference in transition between the control and treatment groups. For those having fenestration before treatment, G4 had a better transition than did G3.

**Conclusions:**

For skeletal class III patients, augmented corticotomy-assisted orthodontic treatment is a promising method of improving alveolar bone dehiscence and fenestration for lower anterior teeth, and it also has the potential to protect both lower and upper anterior teeth against dehiscence.

## Background

Naturally occurring alveolar bone dehiscence and fenestration are common findings in different types of malocclusions [1–5], especially in the anterior region of class III malocclusions [1, 5]. Dehiscence and fenestration may lead to gingival recession and additional bone loss during orthodontic treatment [6–8]. In addition, large amount of labial inclination such as decompensation in class III malocclusions may pose a greater risk of periodontal complications, such as alveolar dehiscence and fenestration and gingival recession [9–12].

Since these disorders can complicate orthodontic treatment by causing gingival recession and additional bone loss, it is important that they be detected before treatment so that they can be treated or prevented. Timock et al. [13] found that the accuracy and reliability of buccal bone height and thickness measurements from CBCT are acceptable and appropriate. Leung et al. [14] found that the diagnostic value of CBCT for detecting buccal defects was high for fenestrations. Sun et al. [15] proposed a CBCT method having a relatively high accuracy to diagnose alveolar dehiscence and fenestration. Nowadays, with CBCT widely used as an orthodontic pretreatment record, alveolar bone dehiscence and fenestration can be easily diagnosed.

Periodontally accelerated osteogenic orthodontics (PAOO) is a combination of bone activation, alveolar augmentation using particulate bone grafting material, and orthodontic treatment [16]. It is believed that PAOO can increase alveolar volume and cover vital root surfaces, which may result in repairing preexisting alveolar dehiscences and fenestrations [16–18]. Some recent studies have reported that alveolar bone thickness may increase after augmented corticotomy-assisted surgical orthodontics [19, 20]. Wang et al. [19] reported that the apical region had a larger amount of alveolar augmentation. Ma et al. [20] applied the “dumpling” technique to augmented corticotomy-assisted orthodontics, and the results showed that both vertical alveolar height and horizontal bone thickness increased in the labial aspect of the anterior mandibular area. Yu et al. [21] reported that alveolar fenestration and bony dehiscence could be successfully addressed after PAOO. However, Wang et al. [19] and Ma et al. [20] did not focus on the changes of alveolar dehiscence and fenestration, and no quantitative description was included in the Yu et al. study [21].

The aim of this study was to evaluate the CBCT-evident changes of alveolar dehiscence and fenestration after augmented corticotomy-assisted orthodontic treatment compared with traditional pre-surgical orthodontics, both quantitatively and qualitatively. The hypothesis was that the changes of alveolar bone dehiscence and fenestration are different after augmented corticotomy-assisted presurgical orthodontic treatment and conventional procedures.

## Methods

### Subjects and samples

Severe class III patients who were to undergo orthognathic surgery from March 2015 to September 2015 were selected from the Department of Oral & Cranio-Maxillofacial Science, Ninth People's Hospital. Twenty-nine patients were selected according to the following inclusion criteria: (1) adult, (2) prepared to undergo orthognathic surgery (bilateral sagittal split ramus osteotomy, Le Fort I osteotomy, or both), (3) mild dental crowding (0–3 mm), and (4) clinical and CBCT examination indicating potential dehiscence and fenestration or thin alveolus and prominent root(s) in the anterior region. After the exclusion criteria—(1) craniofacial syndromes, (2) obvious pathology (cyst or tumor), (3) history of orthodontic treatment, (4) restorations on anterior teeth, (5) defective dentition or supernumerary teeth in the anterior region, or (6) periodontal disease in the anterior region—were applied, the final sample of 17 patients with 204 anterior teeth was selected.

**Fig. 1:**
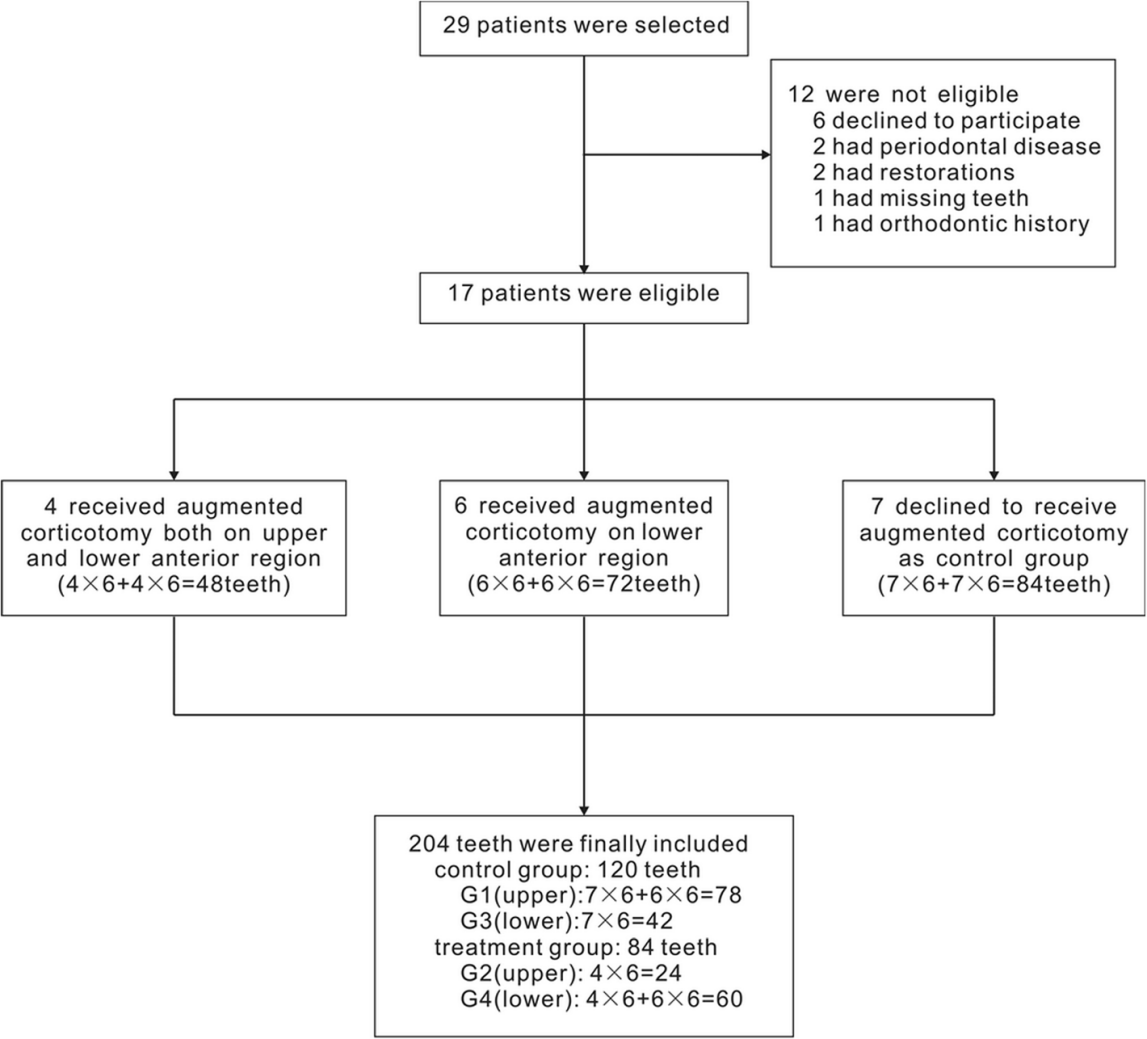
Screening procedure of the selected samples

After being fully informed of the advantages and risks of augmented corticotomy-assisted presurgical orthodontics, seven patients (having 84 teeth) who chose conventional procedures and seven patients (42 upper teeth) who accepted augmented corticotomy in the lower anterior region were allocated to control groups G1 (78 teeth) and G3 (42 teeth), representing upper and lower teeth, respectively. The seven (84 teeth) who chose our decompensation procedures were allocated to treatment groups G2 (24 teeth) and G4 (60 teeth), representing the upper and lower teeth, respectively. Screening procedures of the subjects are shown in Fig. 1. Demographic and pretreatment characteristics of the sample are shown in Table 1. This study was approved by the independent ethics committee of Shanghai Ninth People's Hospital affiliated with Shanghai Jiao Tong University, School of Medicine (IRB NO. 201592). Informed written consent was obtained from each patient and a parent or guardian.

**Table 1:** Demographic and pretreatment characteristics of the sample

Variable	Maxillary		P	Mandibular		P
	G1	G2		G3	G4	
Age, years	20.4 ± 2.1	21.6 ± 3.1	0.424	20.3 ± 2.0	20.9 ± 2.6	0.676
ANB, degree	− 3.6 ± 1.4	− 2.9 ± 2.1	0.508	− 3.3 ± 1.6	− 3.5 ± 1.7	0.821
Overjet, mm	− 1.9 ± 2.0	− 0.8 ± 0.7	0.320	− 1.6 ± 1.1	− 1.7 ± 2.2	0.957
UI-SN, degree	110.1 ± 5.8	115.9 ± 7.9	0.154	111.2 ± 6.1	111.8 ± 7.4	0.881
L1-MP, degree	74.4 ± 6.9	76.6 ± 11.1	0.669	78.7 ± 4.0	72.3 ± 9.2	0.125
Crowding, mm	2.0 ± 0.7	2.1 ± 0.4	0.849	2.0 ± 0.8	2.2 ± 0.6	0.740
Treatment time, years	2.3 ± 0.2	1.6 ± 0.2	0.000	2.3 ± 0.2	2.0 ± 0.3	0.036

### Treatment procedures

**Fig. 2:**
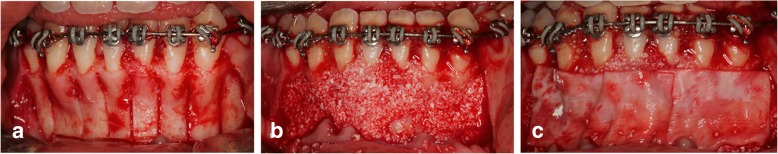
The description of the bone activation and GBR. **a** Performance of selective alveolar decortication. **b** Place bovine inorganic bone over the anterior region. **c** Place collagen membrane over the bone graft material)

Periodontal therapy was conducted for all subjects 2 weeks before the brackets, and wires were engaged. Brackets and tubes were fully bonded to the second molars. The arch-wire sequence involved 0.016-in., 0.018-in., 0.018 × 0.025-in., and 0.019 × 0.025-in. nickel-titanium wires followed by a 0.019 × 0.025-in. stainless steel wire before presurgical orthodontics had been completed [16, 17, 19]. For G1 and G3 dental arches, presurgical orthodontics was carried out at 1-month intervals. For the G2 and G4 dental arches, a 0.016-in. nickel-titanium wire was engaged 1 day before augmented corticotomy, and orthodontic forces were activated at 2-week intervals. Detailed procedures of the augmented corticotomy are illustrated in Fig. 2.

### Image acquisition, processing, and measurement parameters

**Fig. 3:**
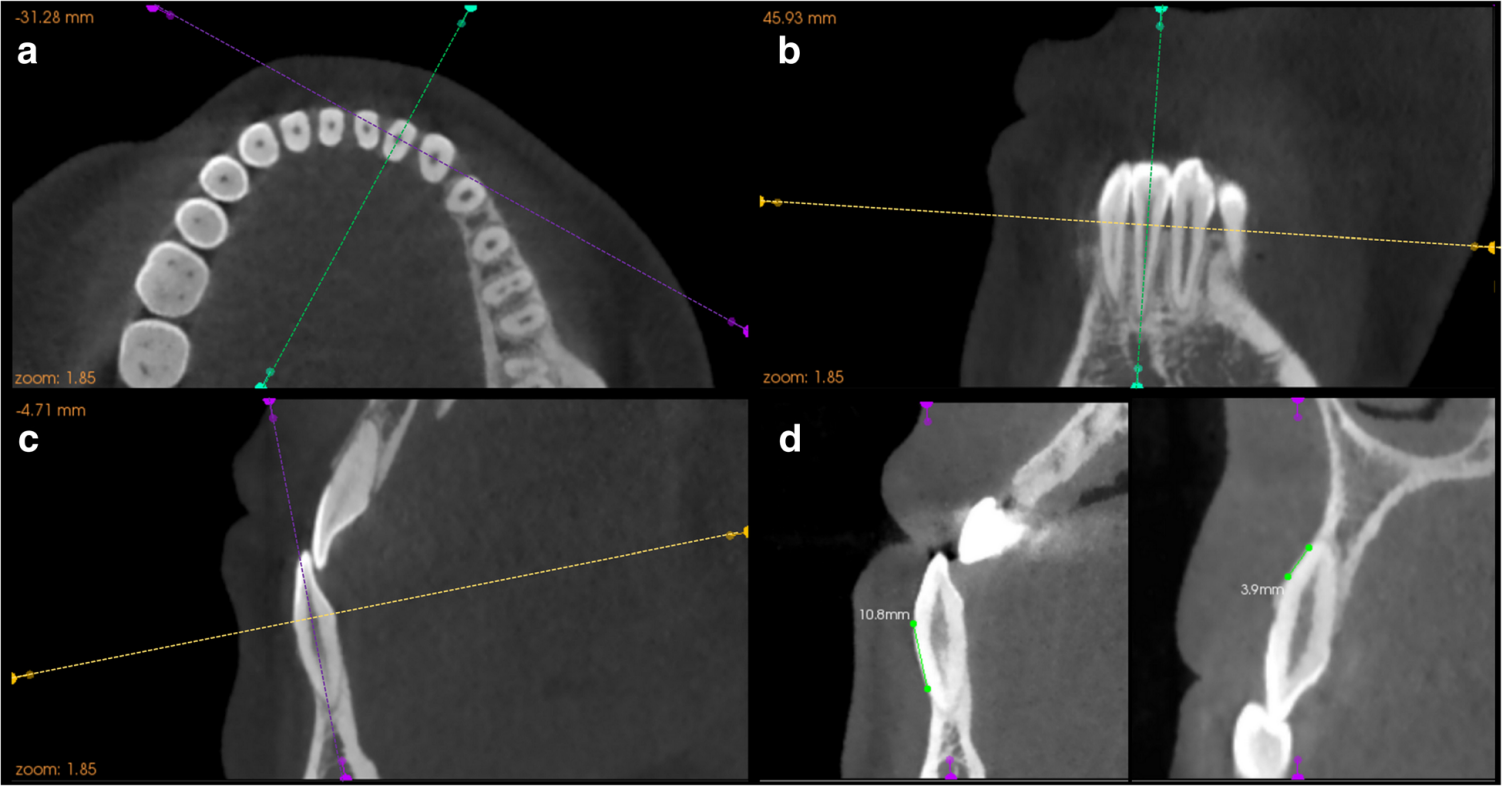
Detailed procedure of locating the measurement plane of the selected tooth. The correlated planes were determined by 3 intersected guide lines with different colors representing the correlated planes, which are yellow for axial plane, green for sagittal plane, and purple for coronal plane. **a** Adjust the location of the axial plane by passing the yellow guideline through the CEJ of the selected tooth in both the coronal and sagittal views, then rotate the purple guideline until the intersecting line is the shortest. **b** Rotate the green guideline until it passes through the root apex and the midpoint of the incisal margin. **c** Rotate the purple guideline until it passes through the root apex and the cusp. **d** To ensure precise and accurate identification of anatomic structures, the largest labiolingual section displayed in the sagittal view was chosen as the measurement plane. A 10.8-mm dehiscence (left) and a 3.9-mm fenestration (right) are shown in the measurement plane

**Fig. 4:**
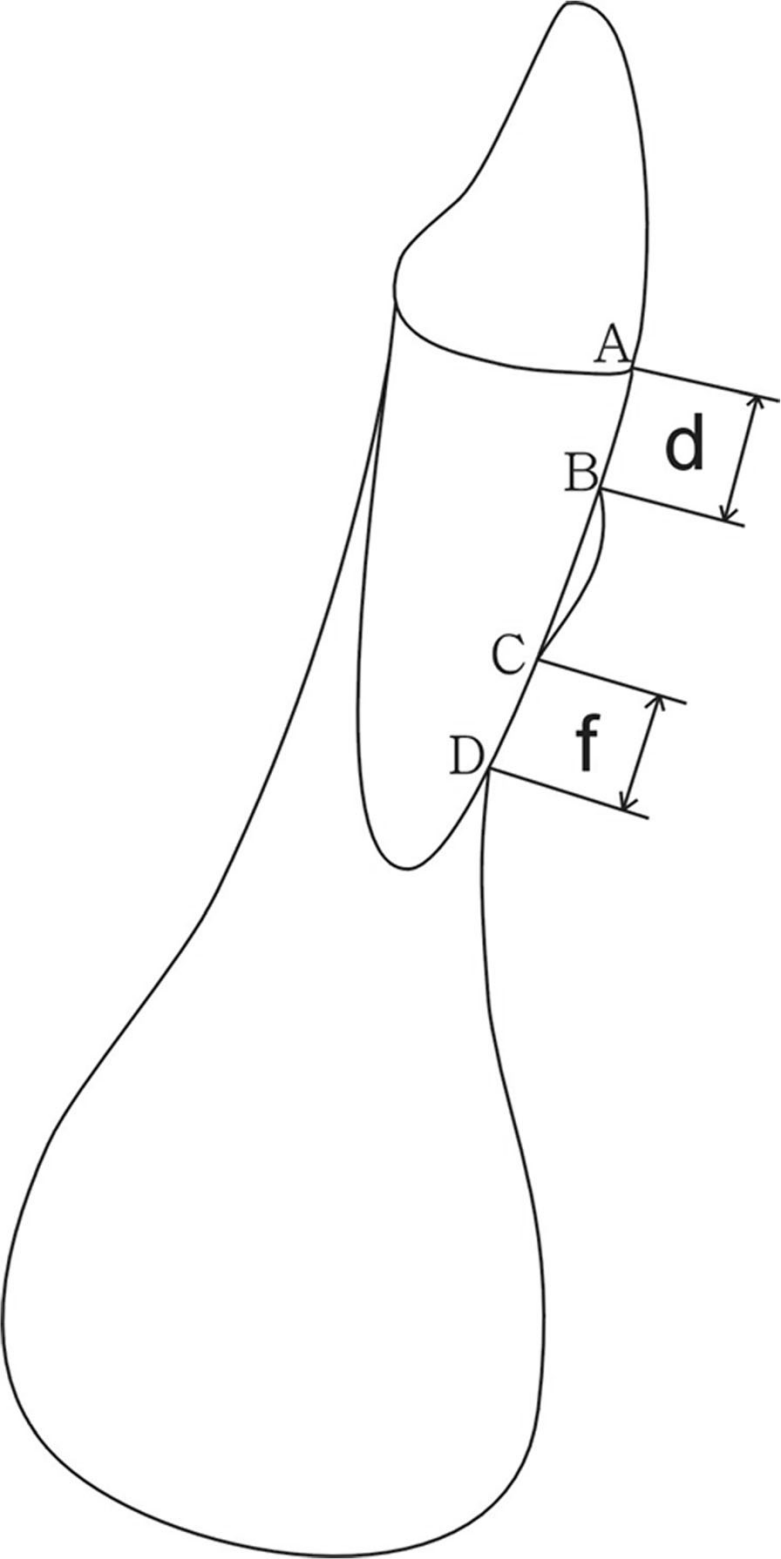
Reference points and measurement variables used in this study

Cone-beam computed tomography images (VG, New Tom, Verona, Italy) were obtained both before presurgical orthodontics and after postsurgical orthodontics (defined as T0 and T1, respectively). The scanning parameters for imaging were 110 kV, 0–20 mA (achieved automatically by setting the grey values at 16 bits), exposure time of 5.4 s, and a 12-in. field of view (F-mode). These settings produced a voxel size of 0.125 mm. The settings were the same as those used for the orthodontic diagnosis and treatment planning in the Department of Oral & Cranio-Maxillofacial Science, Ninth People's Hospital. The acquired CBCT data were imported into the integrated image processing software (Kodak Dental Imaging Software 3D Module V2.4, Eastman Kodak, Rochester, NY, USA). Anterior teeth that fitted our criteria were chosen as study samples, and the largest labiolingual section was defined as the measurement plane. Figure 3 illustrates the detailed protocol of locating the measurement plane. The description of landmarks and measurement variables are modifications of those obtained in the report by Sun et al. [15] (Table 2 and Fig. 4).

**Table 2:** Definition of reference points and variables

Reference points and variables	Definition
Dehiscence	Alveolar bone defect involving an alveolar margin 2 mm or greater and concurrent with a v-shaped BM
Fenestration	A circumscribed defect on the alveolar bone exposing the root, not involving the alveolar crest
A	CEJ at labial side
B	Alveolar crest at labial side
C	The coronal border of a fenestration
D	The apical border of a fenestration
*d* (mm)	The distance between A and B
*d*_0_ (mm)	The distance between A and B at T0
*d*_1_ (mm)	The distance between A and B at T1
*f* (mm)	The distance between C and D
*f*_0_ (mm)	The distance between C and D at T0
*f*_1_ (mm)	The distance between C and D at T1
CPDC (mm)	Critical point of d for dehiscence on CBCT, the value of d more than which the tooth was classified as dehiscence, otherwise it was considered healthy.
CPFC (mm)	Critical point of *f* for fenestration on CBCT, the value of *f* more than which the tooth was classified as fenestration, otherwise it was considered healthy.

According to Sun et al.’s study [15], we set the critical point for dehiscence on the CBCT (CPDC) at 2 mm and the critical point for fenestration on the CBCT (CPFC) at 2.2 mm, which means that when *d* was more than 2 mm on the CBCT, the defect was classified as dehiscence; when *f* was more than 2.2 mm on the CBCT, it was classified as fenestration. To limit experimental bias, dehiscences and fenestrations were re-examined on the CBCT image, the *d* and *f* of all teeth at T0 and T1 were re-measured in 4 weeks, and the resulting mean values were used. All measurements were made by the same operator (L.S.).

Classification of the transition for dehiscence and fenestration after treatment is shown in Table 3.

**Table 3:** The classification of the transition for dehiscence and fenestration after treatment

Transition degree		Definition
*d*_0_ ≤ 2 mm	1	*d*_1_ ≤ 2 mm (maintain)
	2	*d*_1_>2 mm (worsen)
*d*_0_>2 mm	1	*d*_1_ ≤ 2 mm (cure)
	2	2<*d*_1_<*d*_0_ (improve)
	3	*d*_1_ = *d*_0_ (maintain)
	4	*d*_1_>*d*_0_ (worsen)
*f*_0_ ≤ 2.2 mm	1	*f*_1_ ≤ 2.2 mm (maintain)
	2	*f*_1_>2.2 mm (worsen)
*f*_0_>2.2 mm	1	*f*_1_ ≤ 2.2 mm (cure)
	2	2.2<*f*_1_<*f*_0_ (improve)
	3	*f*_1_ = *f*_0_ (maintain)
	4	*f*_1_>*f*_0_ (worsen)

### Statistical analysis

All statistical analyses were performed with the Statistical Package for the Social Sciences (version 16.0; SPSS, Chicago, IL). *d*_0_ and *d*_1_ of all groups were compared by Student’s *t* tests, so were *f*_0_ and *f*_1_ of all groups. Mixed model *t* tests were performed to compare *d*_1_ − *d*_0_ and *f*_1_ − *f*_0_ of both control and treatment groups. Chi-square test and Wilcoxon rank sum test were performed to compare the transition degree of both control and treatment groups. Intra-operator reliability was assessed by calculating the intra-class correlation coefficient (ICC) between measurements collected at both times. The significance level was set at a 2-tailed *P* value of 0.05.

## Results

Table 4 shows the ICC values for *d* and *f* before and after treatment (four continuous variables: *d*_0_, *d*_1_, *f*_0_, and *f*_1_) indicating that our measurements had excellent reliability.

**Table 4:** Intraoperator reliability for *d* and *f* measurements before treatment (T0) and after treatment (T1) by means, standard deviations, and ICC

	T1			T2		
	Measurement 1	Measurement 2		Measurement 1	Measurement 2	
*n* = 204	Mean ± SD (mm)	Mean ± SD (mm)	ICC	Mean ± SD (mm)	Mean ± SD (mm)	ICC
*d*	3.51 ± 3.02	3.55 ± 3.08	0.995	3.77 ± 3.16	3.71 ± 3.10	0.992
f	1.56 ± 1.79	1.60 ± 1.85	0.994	1.07 ± 1.57	1.05 ± 1.53	0.997

Table 5 shows the changes of *d* and *f* after treatment. For G1, there was a significant increase in *d* (*P* < 0.01) and an insignificant increase in *f*. For G2, there was an insignificant decrease in both *d* and *f*. For G3, there was a significant increase in *d* (*P* < 0.01) and a significant decrease in *f* (*P* < 0.01). For G4, there was a significant decrease in both *d* and *f* (*P* < 0.01).

**Table 5:** Changes of d and f after treatment

	G1		G2		G3		G4	
	Mean ± SD	*P*	Mean ± SD	*P*	Mean ± SD	*P*	Mean ± SD	*P*
*d*_0_	1.873 ± 1.841	0.009	3.346 ± 2.198	0.062	5.283 ± 3.199	0.005	4.520 ± 3.440	0.009
*d*_1_	2.763 ± 3.414		2.546 ± 1.214		6.826 ± 2.691		3.320 ± 2.015	
*f*_0_	1.377 ± 1.610	0.240	1.008 ± 1.398	0.350	1.674 ± 1.795	0.002	2.015 ± 2.143	0.000
*f*_1_	1.659 ± 2.227		0.738 ± 0.834		0.888 ± 1.540		0.527 ± 0.207	

Table 6 shows the comparison of changes of *d* (*d*_1_ − *d*_0_) and *f* (*f*_1_ − *f*_0_) between the control and treatment groups. For the upper teeth, *d*_1_ − *d*_0_ was statistically different between both groups (*P* < 0.01) while *f*_1_ − *f*_0_ was not statistically different. For the lower teeth, *d*_1_ − *d*_0_ was statistically different between both groups (*P* < 0.01) while *f*_1_ − *f*_0_ was not statistically different.

**Table 6:** Comparison of *d*_1_ − *d*_0_ and *f*_1_ − *f*_0_ for samples between control and treatment groups

	G1		G2			G3		G4		
	Mean	SD	Mean	SD	*P*	Mean	SD	Mean	SD	*P*
*d*_1_ − *d*_0_	0.89	2.928	− 0.8	1.551	0.002	1.543	3.339	− 1.2	3.433	0.000
*f*_1_ − *f*_0_	0.282	2.105	− 0.271	1.390	0.141	− 0.786	1.54	− 1.488	2.172	0.059

Table 7 compares the degree of transition for dehiscence and fenestration between the control and treatment groups. For teeth having no dehiscence before treatment, G2 and G4 had a better transition than did G1 or G3 (*P* < 0.05). For those having dehiscence before treatment, G4 had a better transition than did G3 (*P* < 0.01), but there was no statistically significant difference in the transition between G1 and G2. For teeth having no fenestration before treatment, there was no statistically significant difference in the transition between the control and treatment groups. For those having fenestration before treatment, G4 had a better transition than did G3 (*P* < 0.01), but there was no statistically significant difference between G1 and G2.

**Table 7:** Comparison of transition degree for dehiscence and fenestration between control and treatment groups

Transition degree			G1	G2	*P*	G3	G4	*P*
Dehiscence	*d*_0_ ≤ 2	1	41	2	0.0412	1	10	0.0205
		2	10	3		7	6	
	*d*_0_ > 2	1	6	7	0.1193	2	7	0.0039
		2	3	7		7	20	
		3	1	1		2	1	
		4	11	4		23	16	
Fenestration	*f*_0_ ≤ 2.2	1	41	19	0.126	24	36	0.080
		2	10	1		1	1	
	*f*_0_ > 2.2	1	6	3	0.208	11	23	0.002
		2	7	0		4	0	
		3	0	0		0	0	
		4	8	1		2	0	

## Discussion

The detection of alveolar bone dehiscence and fenestration in vivo can be achieved in three possible ways: inspecting during mucogingival surgery, exploring with the periodontal probe, and radiograph. Since patients undergoing periodontal surgery are very few and carefully selected, the first method cannot be widely applied. Using a periodontal probe and taking traditional X-rays are unreliable and unsatisfactory. Timock et al. [13] found strong agreement between CBCT and direct measurements for buccal bone height and thickness, which speaks for the accuracy and reliability of CBCT in measuring those parameters. Researches have shown that computed tomography and CBCT are highly effective in detecting artificially created bony defects and naturally occurring alveolar dehiscences and fenestrations on skulls [14, 22–25]. Leung et al. [14] reported that under a 0.38-mm voxel size, the diagnostic value of CBCT for detecting buccal defects was high for fenestrations: both sensitivity and specificity were about 0.80; for dehiscences, the specificity was high at 0.95, but the sensitivity was low at 0.40. Our previous study [15] showed that under a 0.125-mm voxel size, while using the best critical points (CPDC = 2.2 mm, CPFC = 2.2 mm), both sensitivity and specificity for dehiscence and fenestration were acceptable (both about 0.8). The PV+ value for detecting dehiscence was 0.84, and the PV− value for detecting dehiscence and fenestration was 0.79 and 0.98, respectively, which means that 84% of the detected dehiscences truly existed and most of the healthy teeth diagnosed by CBCT were truly healthy. On the other hand, the PV+ value for detecting fenestration was 0.21, meaning that when a fenestration was detected, it was a true fenestration about 20% of the time. By using our CBCT settings and methods, although there was a systematic overestimation of the CBCT measurements, its diagnostic value in detecting alveolar bone dehiscences and fenestrations was still acceptable. It can be concluded that at present the only satisfactory method of detecting alveolar bone dehiscence and fenestration in vivo before treatment is the CBCT method.

This study showed a higher reliability of measuring the vertical diameter of dehiscences and fenestrations by CBCT, with ICC values of 0.992 and 0.997. The time interval between the first and second measurements was 4 weeks. Our results were similar to that reported by Leung et al. [14] (0.891–0.994) and Sun et al. [15] (0.994–0.0996).

For class III malocclusion, presurgical orthodontic decompensation requires uprighting the proclined maxillary incisors and retroclining the mandibular incisors to more normal axial inclinations. Large amounts of dental decompensation might be associated with a higher tendency to develop gingival recessions [26–28]. However, there are only a few studies on alveolar bone change after orthodontic treatment. Wehrbein et al. [29] evaluated the alveolar bone of a deceased patient who had undergone orthodontic treatment and found severe bone loss on the labial and lingual cortical plates. Lee et al. [9] evaluated the alveolar bone loss around lower incisors incurred during surgical orthodontic treatment in 25 individuals with mandibular prognathism. They concluded that excessive forward movement of lower incisors during presurgical orthodontic treatment could cause alveolar bone loss. Our results showed the same tendency: After traditional treatment, dehiscence of both upper and lower anterior teeth worsened, while fenestration of lower anterior teeth improved. For lower anterior teeth, because root lingual torque control might mitigate the prominence of the apexes, fenestration improved.

The aim of our study was to evaluate on CBCT the changes of alveolar dehiscence and fenestration after augmented corticotomy-assisted orthodontic treatment compared with traditional pre-surgical orthodontics, both quantitatively and qualitatively. Our results suggest that, after augmented corticotomy-assisted orthodontic treatment, both dehiscence and fenestration improved for the lower anterior teeth. Compared with traditional treatment, the augmented corticotomy-assisted orthodontic treatment showed more healing effect for dehiscences than for fenestration in both upper and lower anterior teeth. Table 7 indicates that, compared with traditional treatment, augmented corticotomy-assisted orthodontic treatment has the potential to protect both upper and lower healthy anterior teeth against dehiscence. The results also suggest that augmented corticotomy-assisted orthodontic treatment had a healing effect on lower anterior teeth region both for dehiscence and fenestration.

Just as the most probable etiologic cause of alveolar dehiscence and fenestration is a combination of prominent roots and a thin alveolar bone plate, the most probable mechanism for healing is the combination of root control and the augmented bone graft. As previously mentioned, recent studies have reported that an increase in alveolar bone thickness which covers the root surface might be explained by the same mechanism. That is, first, either the bone graft material augmented the hard tissue overlying the root surface or it eliminated instantly the pre-existing defect. Then, the increased alveolar volume and a more structurally complete periodontium allowed us to move the teeth safely. After decompensation and torque control during presurgical orthodontics, the position and inclination of the teeth were controlled and the roots were set in the center of the alveolar process.

However, one important question remains: Can true periodontal regeneration be achieved on the root surface? Is the bone augmentation seen on CBCT real bone tissue, or just bone material, or a mixture of both? Unlike Wilcko’s [16] study, we did not perform the re-entrance to obtain the biopsy, and the duration was relatively short, especially for the treatment groups. Experimental animal studies and long-term follow-up are required in the future. Our study also had other limitations. Our subjects were all skeletal class III malocclusions, and we collected only anterior teeth, because alveolar dehiscences and fenestrations are most common in the anterior region of class III malocclusions. In addition, our sample size was not large enough. More subjects having different kinds of malocclusions should be accumulated, and both anterior and posterior teeth should be included in the future.

## Conclusions

For skeletal class III patients, augmented corticotomy-assisted orthodontic treatment is a promising method to improve the alveolar bone dehiscence and fenestration of lower anterior teeth, and it also has the potential to protect both lower and upper anterior teeth from dehiscence.

## Abbreviations

CBCT: Cone-beam computed tomography
CPDC: Critical point for dehiscence on the CBCT
CPFC: Critical point for fenestration on the CBCT
PAOO: Periodontally accelerated osteogenic orthodontics
